# Supplementary figures and images for: Glycosylated extracellular mucin domains protect against SARS-CoV-2 infection at the respiratory surface
Source: PLoS Pathog. 2023 Aug 10;19(8):e1011571. doi: 10.1371/journal.ppat.1011571 (PMC10464970; doi:10.1371/journal.ppat.1011571)

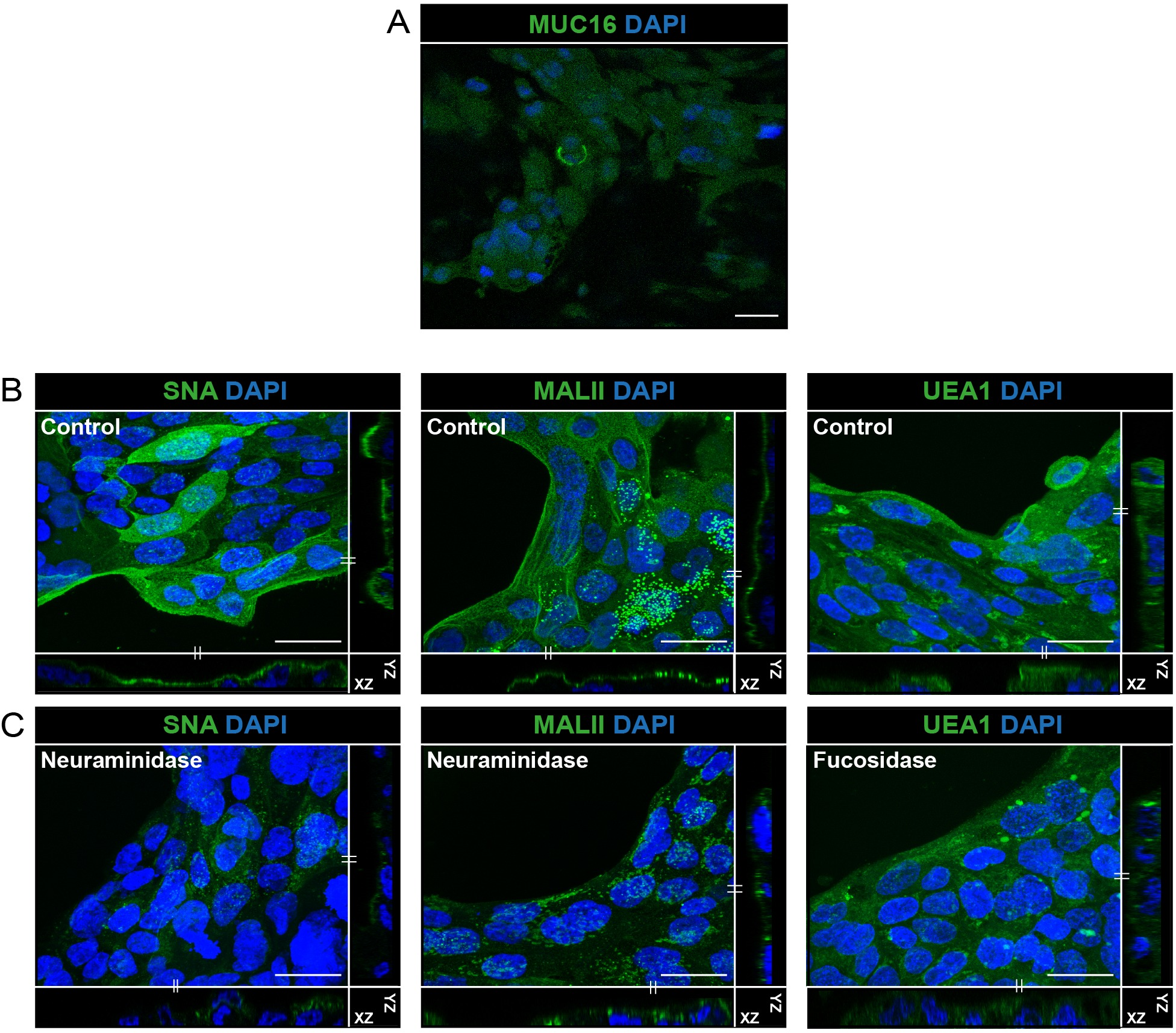

Supplement: S1 Fig — (A) Immunofluorescence confocal microscopy of 4-days grown Calu-3 cells revealed very limited expression of MUC16 (α-MUC16 ED, green). (B) Immunofluorescence confocal microscopy images for α-2,6 sialic acid (SNA, green) and α-2,3 sialic acid (MALII, green) levels after neuraminidase treatment and fucose (UEAI, green) after fucosidase treatment of Calu-3 cells. Nuclei were stained with DAPI (blue). White scale bars represent 20 μm. (TIF) [file ppat.1011571.s001.tif]

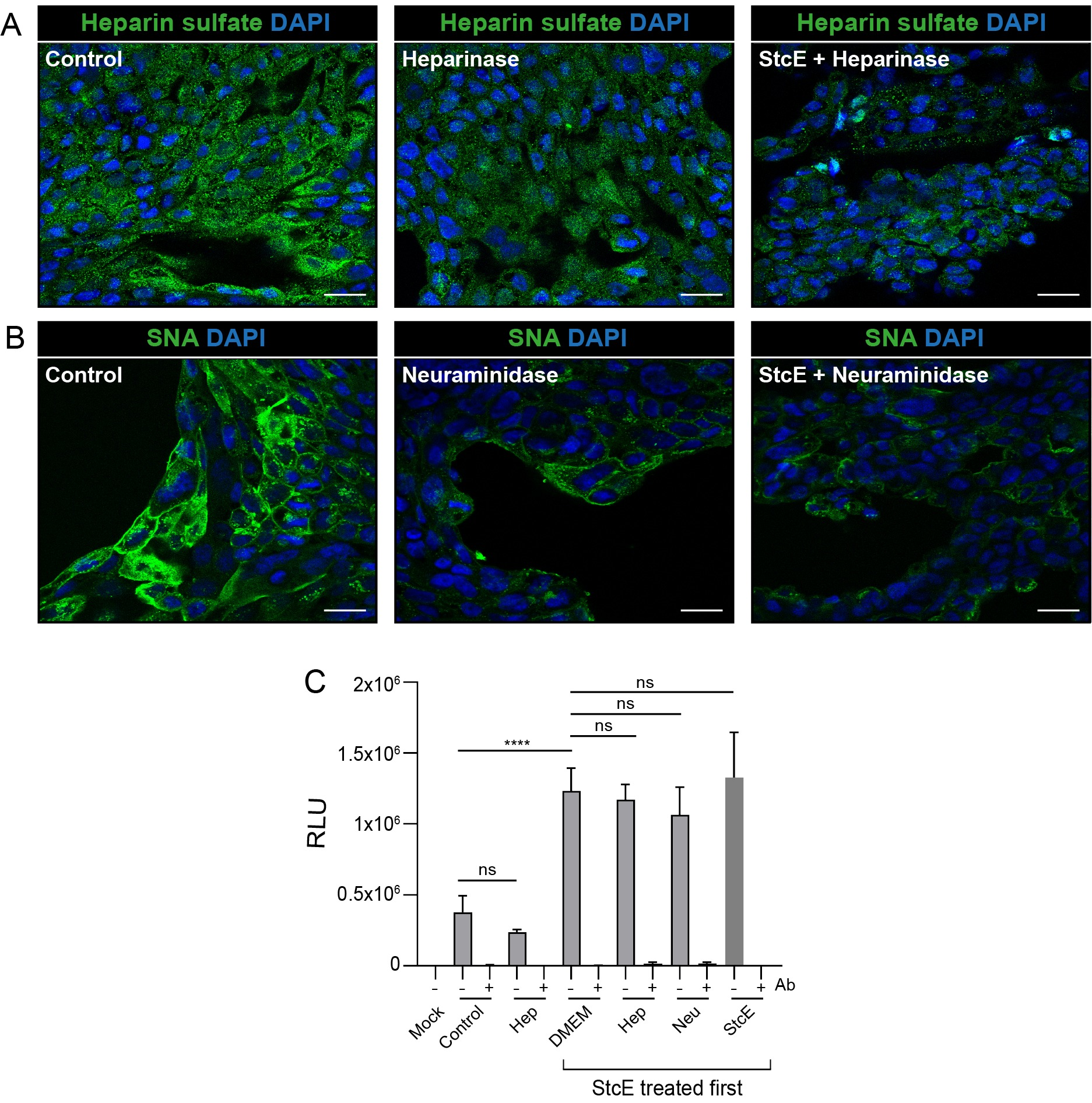

Supplement: S2 Fig — (A) Confocal microscopy image showing levels of heparin sulfate (F69-3G10, green) in control, heparinase-treated and StcE/heparinase-treated Calu-3 cells. (B) Confocal microscopy image showing levels of α-2,6 sialic acid (SNA, green) control, neuraminidase-treated and StcE/neuraminidase-treated Calu-3 cells. Nuclei were stained with DAPI (blue). White scale bars represent 20 μm. (C) Luciferase quantification of viral infection of Calu-3 cells were treated with StcE for 3 h at 37°C followed by heparinase or neuraminidase for an additional 3 h at 37°C and infection with VSVΔG-Rluc*SARS2-Spike without or with monoclonal antibody (mAb) against SARS2-Spike. No significant changes in RLU values were observed in any of the cases. Represented values are the mean ± SEM of three biological replicates performed in triplicate. Statistical analysis was performed by repeated measures one way-ANOVA with Dunnett’s post-hoc test. p > 0.05 [ns, not significant], p<0.05 [*], p<0.01 [**], p<0.001 [***], p<0.0001 [****]. (TIF) [file ppat.1011571.s002.tif]

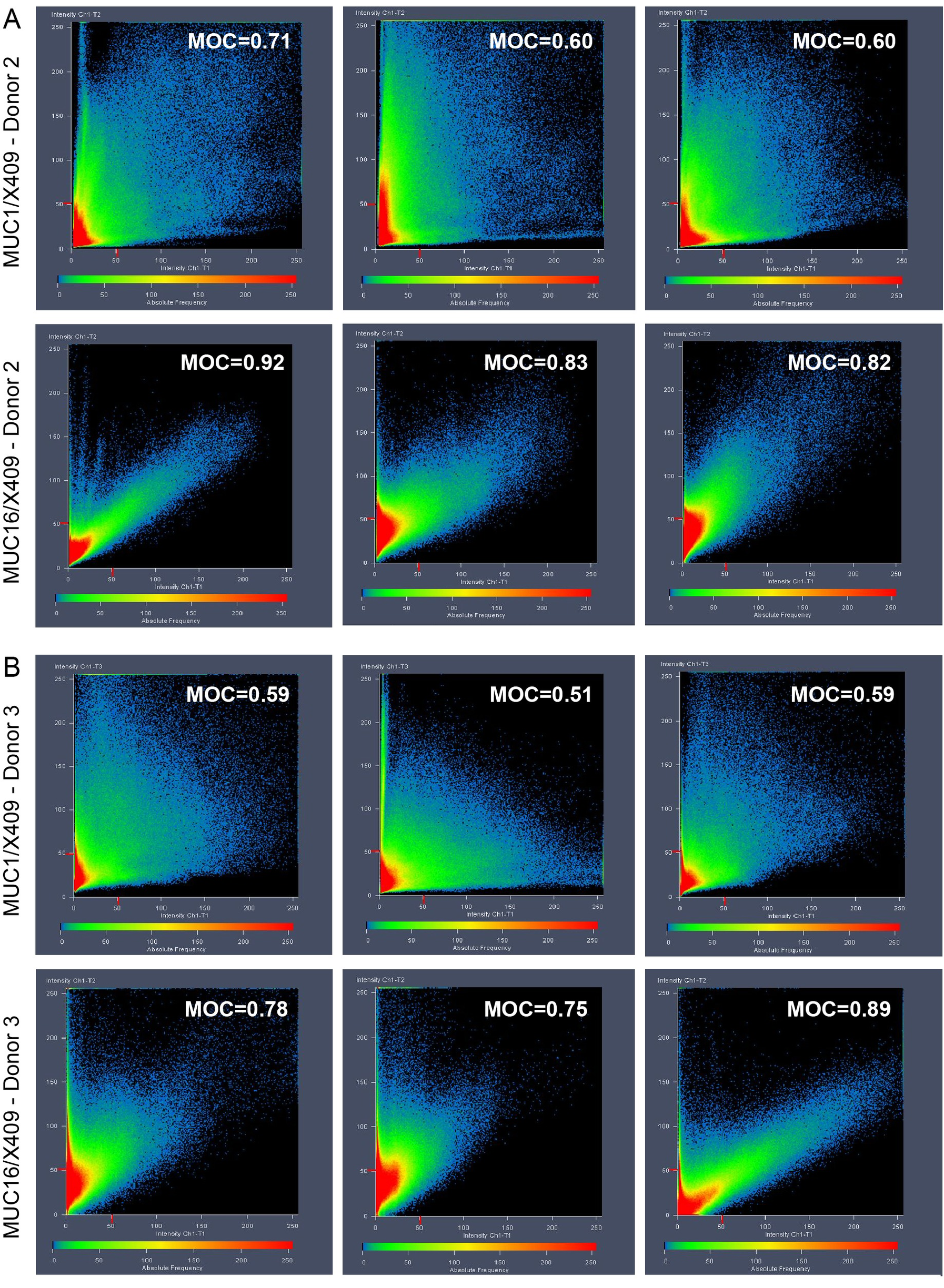

Supplement: S3 Fig — Organoid-derived airway ALI cultures were stained for MUC1 or MUC16 in combination with X409 as depicted in Fig 4D. Colocalization of MUC1 and MUC16 with X409 was quantified cultures from dono1 2 (A) and donor 3 (B) by plotting Mander’s overlap coefficient using ZEN software. The summary of this quantification is depicted in Fig 4E. (TIF) [file ppat.1011571.s003.tif]
