# Supplementary material for: Glycosylated extracellular mucin domains protect against SARS-CoV-2 infection at the respiratory surface
Source: PLoS Pathog. 2023 Aug 10;19(8):e1011571. doi: 10.1371/journal.ppat.1011571 (PMC10464970; doi:10.1371/journal.ppat.1011571)
Supplement: S1 Methods — (DOCX) [file ppat.1011571.s004.docx]

**Supplementary methods**

**Production of pseudotyped vesicular stomatitis virus (VSV) and SARS2-Spike pseudotyped VSV virus**

BHK-21 cells were co-transfected with the plasmids pVSVΔG-GFP (green fluorescent protein reporter) or pVSVΔG-Rluc (*Renilla reniformis* luciferase reporter) and the four assembly plasmids encoding the VSV-N, -P, -G and -L proteins. The VSVΔG-GFP and Rluc virus stocks were harvested and added to BHK-21 cells that were transfected with pCAGGS expression vector encoding VSV-G glycoprotein for the production of VSV-G pseudotyped VSV-GFP and VSV-G pseudotyped VSV-Luc virus. After 48 h, supernatant containing VSV-G pseudotyped VSV-GFP or VSV-G pseudotyped VSV-Luc viral particles were harvested and used for the production of SARS2-Spike pseudotyped VSV-GFP or SARS2-Spike pseudotyped VSV-Luc respectively according a previously described method (1).

For the production of SARS-CoV-2 pseudotyped virus, HEK-293T cells were transfected with a pCAGGS expression vector encoding SARS-CoV-2 Spike carrying an 18-a.a. cytoplasmic tail truncation (SARS2-S-d18-Flag). Two days post-transfection, cells were infected with the VSV-G pseudotyped VSV-GFP or VSV-G pseudotyped VSV-Luc at a multiplicity of infection (MOI) of 1. After 4 h of infection, infection medium was removed and washed thoroughly to remove surface-attached VSV pseudovirus. Twenty-four hours later, supernatants containing SARS2-Spike pseudotyped VSV particles (SARS2-S pseudotyped VSV-GFP and SARS2-S pseudotyped VSV-Luc) were harvested and stored at -80°C until use. For the virus neutralization assay, monoclonal antibody (mAb) REGN10933 against SARS2-Spike (developed in the group of Berend Jan Bosch) was used at a final concentration of 1 μg/ml in DMEM supplemented with 1% FCS (Gibco), 100 U/ml Penicillin and 100 µg/ml Streptomycin. Diluted mAbs were incubated with an equal volume of SARS2-S pseudotyped VSV-GFP or SARS2-S pseudotyped VSV-Luc viral particles for 1 hour at room temperature, followed by inoculation on confluent Calu-3 cells in a 96-well plate and incubation at 37°C for 18-20 h. Luciferase activity was measured on a Berthold Centro LB 942 plate luminometer using the *Renilla* luciferase substrate coelenterazine (Promega). The percentage of infectivity was determined as a ratio of luciferase signal in the absence of mAbs normalized to luciferase signal in the presence of mAb.

**Production of authentic SARS-CoV-2 virus stock**

Briefly, cells were infected at a MOI of 0.01 and incubated for 48-72 h. Collected supernatant was cleared by centrifugation before being additionally cleared using a 0.45 μM low protein binding filter (Millipore) to remove mucus debris produced by the cells. The medium was exchanged three times with Opti-MEM I (1X) + GlutaMAX (Gibco) using an Amicon Ultra-15 column (100 kDa cutoff). After three exchanges, the purified virus was transferred to a new 50 ml tube and the Amicon Ultra-15 column was washed with 1 ml Opti-MEM I (1X) + GlutaMAX (Gibco), adding each wash to the tube containing the purified virus preparation until the volume in the purified virus stock was equal to the original volume of culture supernatant. Purified virus was stored at −80°C in aliquots. Stock titers were determined by plaque assay. Briefly, 10-fold serial dilutions were performed in 2 ml Opti-MEM I (1X) + GlutaMAX (Gibco). One ml of each virus dilution was added to monolayers of Calu-3 cells in the same medium in a 12 well plate. Cells were incubated at 37°C for 1 hr and then overlaid with 1.2% Avicel (FMC biopolymers) in Opti-MEM I (1X) + GlutaMAX (Gibco) for 72 hours. Next, cells were washed once in PBS, fixed in formalin, permeabilized in 70% ethanol and washed in PBS again. Cells were blocked in 3% BSA (bovine serum albumin; Sigma) in PBS, stained with mouse anti-nucleocapsid (Sino biological; 1:1000) in PBS containing 0.1% BSA, washed three times in PBS, then stained with goat anti-mouse Alexa Fluor 488 (Invitrogen; 1:2000) in PBS containing 0.1% BSA and then washed three times in PBS. All staining steps were performed at room temperature for one hour. Plates were scanned on the Amersham Typhoon Biomolecular Imager (channel Cy2; resolution 10 µm; GE Healthcare). All work with infectious SARS-CoV-2 was performed in a Class II Biosafety Cabinet under BSL-3 conditions at Erasmus Medical Center.

**Confocal microscopy**

For staining of the α-MUC1 ED, α-MUC1 SEA, α-MUC4 ED, α-MUC16 ED, α-MUC5AC, SNA, MALII, UEA1, ACE2, and Spike, fixed cells on coverslips were permeabilized in binding buffer containing 0.1% saponin (Sigma) and 0.2% BSA (Sigma) in DPBS for 30 min. After permeabilization, cells were washed two times with DPBS and incubated onto 50-μl drops containing 214D4 antibody (CD227, Nordic MUbio) for α-MUC1 ED, 232A1 antibody (a kind gift from Dr. John Hilkens, AVL Amsterdam) for α-MUC1 SEA, α-MUC4 ED (8G7; sc-53945, Santa Cruz Biotechnology), α-MUC5AC (ab198294, Abcam), α-MUC16 ED (a kind gift from Ulla Mandel, University of Copenhagen, Denmark), biotinylated-SNA (B-1305-2; Vector Lab), biotinylated-MALII (B-1265-1; Vector Lab), biotinylated-UEA1 (B-1065-2; Vector Lab), ACE2 (ab272690, Abcam), Heparin sulphate (370260-S; Amsbio) diluted 1:100 and Fc-tagged SARS2-S1B-Fc (a kind gift from Berend Jan Bosch, Utrecht University) diluted 1:164 in the binding buffer on parafilm for 1 h at RT. For staining of the MUC1 CT domain, cells on coverslips were permeabilized in DPBS containing 0.2% Triton X-100 (Merck) for 10 min. After permeabilization, cells were blocked with 1% BSA and 22.5 mg/ml glycine in PBST (DPBS + 0.1% Tween 20 [Sigma]) for 30 min and washed three times with DPBS. Cells were incubated onto 50-μl drops of the α-MUC1-CT antibody (ab80952, Abcam) diluted 1:100 with 1% BSA in PBST on parafilm for 1 h at RT. For non-permeabilization microscopy, the cell permeabilization step was omitted and primary antibody was used in 0.2% BSA in DPBS for 1 h at RT. After removing the primary antibody, 3 washing steps were performed. The coverslips were further incubated with the secondary antibodies Alexa Fluor 488-conjugated goat α-mouse IgG (1:100; A11029, Thermo Fisher), Alexa Fluor 568-conjugated goat α-mouse IgG (1:100; A11031, Thermo Fisher), Alexa Fluor 647-conjugated goat α-mouse IgG (1:100; ab150115, Abcam), Alexa Fluor 488-conjugated goat α-rabbit IgG (1:100; A11034, Thermo Fisher), Alexa Fluor 488-conjugated goat α-Armenian hamster IgG (1:100, ab173003, Abcam), Alexa Fluor 594-conjugated goat α-human IgG (1:400; A-11014, Thermo Fisher), Streptavidin-488 (1:100; A6374, Thermo Fisher) and 568 (1:100; S11226, Thermo Fisher) and DAPI at 2 μg/ml (D21490, Invitrogen) for 1 h. Coverslips were washed 3 times with DPBS, washed a final time with MilliQ, dried, embedded in Prolong diamond mounting solution (Thermo Fisher), and allowed to solidify overnight at RT. For spike binding experiments, 2.5 ug/ml purified Fc-tagged spike protein of SARS-CoV-2 (SARS2-S1B-Fc) was added to Calu-3 cells at 4°C for 1 h after StcE and E447D treatment. Cells were washed thoroughly to remove unbound spike before fixation and followed the non-permeabilization protocol for staining.

For costaining of the α-MUC1 ED and ACE2, fixed cells on coverslips were permeabilized in 70% absolute ethanol (Merck) for 10 min. After permeabilization, cells were washed two times with DPBS and then blocked with 0.6% BSA in DPBS for 30 min. Cells were incubated onto 75-μl drops containing 214D4 antibody (CD227, Nordic MUbio) diluted 1:100 for MUC1-ED and ACE2 antibody (AF933, R&D Systems) diluted 1:200 with 0.6% BSA in DPBS on parafilm overnight at 4°C. Afterwards three washing steps were performed. The coverslips were further incubated with the secondary antibodies Alexa Fluor 488-conjugated rabbit α-goat IgG (1:500; A11078, Invitrogen), Alexa Fluor 568-conjugated donkey α-mouse IgG (1:100; A10037, Thermo Fisher) and DAPI at 2 μg/ml (D21490, Invitrogen) for 1 h. Coverslips were washed 3 times with DPBS, washed a final time with MilliQ, dried, embedded in Prolong diamond mounting solution (Thermo Fisher), and allowed to solidify overnight at RT. For all confocal experiments, incubation with only the secondary antibody were used as negative controls.

A Leica SPE-II confocal microscope (Leica Microsystems, Wetzlar, Germany) was used to acquire single plane images with a 40X objective (NA 1.3, HCX PLANAPO oil) controlled by Leica LAS AF software with default factory settings. A quad band dichroic was used, allowing diode laser wavelengths 405, 488, 561 nm lines to pass and fluorescent signal to enter the prism to sequentially detect DAPI, Alexa Fluor 488, Alexa Fluor 568/594, Alexa Fluor 647. Images stacks were collected using a 63X oil immersion objective in bidirectional mode (average 16). Sequential laser illumination through a quadband dichroic (405, 488, 561, and 647) was used in combination with emission factor default spectral detection of DAPI, Alexa Fluor 488, Alexa Fluor 568/594, and Alexa Fluor 647, respectively. XZ and YZ Orthogonal views of 30-pixel thickness are shown generated in either Imaris (Oxford Instruments version 8.2, Belfast, UK). Acquired images were processed in Leica Application Suite X (LAS X; Leica). Maximum intensity projections are shown, as well as representative slices from the image series. Final outlining of the figures was performed in Adobe Illustrator (Adobe Inc., San Jose, USA).­

Fluorescent spike signal was quantified by ImageJ software (2). A segmented line (line width=10) was outlined along the edge of the cell island to select the region of interest (ROI) in control, StcE and E447D treated Calu-3 cells. The sum of all pixels in the ROI was measured by Raw Integrated Density (RawIntDen) and the bar diagram was represented as RawIntDen/length (µm). Three random regions were selected, and three independent experiments were performed.

Spinning disk imaging was performed on an Olympus SpinSR10 system equipped with a Yokogawa W1-SoRa spinning disk mounted on a IX83 stand with an ORCA Flash 4.0 camera (Olympus, Leidendorp, the Netherlands). The system was run in confocal mode using a 60 x Uplan Apo OHR objective (NA 1.5) and multiband dichroic to sequentially illuminate 405, 488 and 561 nm laserlines, emission filter wheels set to detect 447/60, 525/50 and 617/73 bandwidths for DAPI, Alexa488 and Alexa568 respectively. Images were deconvolved using maximum likelihood estimation in 5 iterations in CellSense Dimension (Olympus) and intensity adjusted.

**PLA assay for MUC1 and ACE2**

For staining of the α-MUC1 ED and ACE2, fixed cells on coverslips were permeabilized in 70% absolute ethanol (Merck) for 10 min. After permeabilization, cells were washed two times with DPBS. Cells were blocked onto 40-μl drops Duolink Blocking Solution (DUO82007, Merck) for 60 minutes at 37°C on parafilm in a humidity chamber. Afterwards, cells were incubated onto 60-μl drops containing ACE2 antibody (AF933, R&D Systems) diluted 1:200 and 214D4 antibody (CD227, Nordic MUbio) for α-MUC1 ED or 232A1 antibody (a kind gift from Dr. John Hilkens, NKI-AVL Amsterdam) for α-MUC1 SEA diluted 1:100 with Duolink Antibody Diluent (DUO82008, Merck) overnight at 4°C. Coverslips were washed 4 times with 1 mL Wash Buffer A (DUO82046, Merck) while shaking. Next, coverslips were incubated onto 30 μl drops of Duolink PLA probe anti-goat PLUS (DUO82003, Merck) and Duolink PLA probe anti-mouse MINUS (DUO82004, Merck) diluted 1:10 in Duolink Antibody Diluent for 60 minutes at 37°C and washed as above. Afterwards, a ligation step was performed with 30 μl drops of Ligase (DUO82029, Merck) diluted 1:40 in 1x Ligation Buffer (DUO82009, Merck) for 60 minutes at 37°C and washed as above. From this moment onwards, coverslips were protected from light. An amplification step was performed with 30 μl drops of Polymerase (DUO82030, Merck) diluted 1:40 – 1:80 in 1x Duolink Amplification Red (DUO82011, Merck) for 100 minutes at 37°C. Cells were washed 1 time before incubating with secondary antibodies Alexa Fluor 488-conjugated rabbit α-goat IgG (1:500; A11078, Invitrogen), Alexa Fluor 647-conjugated donkey α-mouse IgG (1:100; 715-605-151, Jackson ImmunoResearch) and DAPI at 2 μg/ml (D21490, Invitrogen) for 30 minutes at 37°C. Coverslips were washed 2 times with Wash buffer A, 1 time with 0.01x Wash Buffer B (DUO82048, Merck), dried by air, embedded in Prolong diamond mounting solution (Thermo Fisher), and allowed to solidify overnight at RT before microscopy analysis.

References

1. Widjaja I, Wang C, van Haperen R, Gutiérrez-Álvarez J, van Dieren B, Okba NMA, et al. Towards a solution to MERS: protective human monoclonal antibodies targeting different domains and functions of the MERS-coronavirus spike glycoprotein. Emerg Microbes Infect. 2019;8(1):516–30.

2. Schindelin J, Arganda-Carrera I, Frise E, Verena K, Mark L, Tobias P, et al. Fiji - an Open platform for biological image analysis. Nat Methods. 2009;9(7).
