# Supplementary material for: Glycosylated extracellular mucin domains protect against SARS-CoV-2 infection at the respiratory surface
Source: PLoS Pathog. 2023 Aug 10;19(8):e1011571. doi: 10.1371/journal.ppat.1011571 (PMC10464970; doi:10.1371/journal.ppat.1011571)
Supplement: Attachment — Submitted filename: Rebuttal PLOS Pathogens_310523.docx [file ppat.1011571.s005.docx]

We thank the reviewers for their constructive comments. We are excited that we were able to add several interesting new figures based on their suggestions. The additions to the manuscript include

spike binding to ALI cultures after mucinase treatment, quantification of MUC1 and MUC16 colocalization with X409, dotblot analysis of MUC1 after enzymatic treatment, proximity ligation assays that demonstrate ACE2/MUC1 interaction in Calu-3, expression and localization of MUC1 and MUC16 in different cell types in airway cultures and new schematic models to summarize the findings. Below we included a point-by-point response to the reviewers. We think the manuscript has been greatly improved by this review process for which we thank you. We hope that this revised manuscript is acceptable for publication in PLOS Pathogens.

**Part I - Summary**
Please use this section to discuss strengths/weaknesses of study, novelty/significance, general execution and scholarship.

Reviewer #1: Understanding host-pathogen interactions at the cellular surface are essential for improving our knowledge surrounding infection processes and host-defences. This expert team asks the important question as to whether the cell-surface and gel-forming mucins of the respiratory mucosa aid or hinder the ability for SARS-CoV-2 to infect cells. Using appropriate mucinases, fucosinases, neuroaminidases, and antibody controls the authors conclusively show that the cell-surface MUC1 extracellular domain provided a barrier-type role and aided in limiting SARS-CoV-2 infection of the underlying cell. When MUC1 was expressed in it’s natural form, less SARS-CoV-2 was able to gain access to ACE2 (the virus receptor), while removal of the extracellular domain of MUC1 enhanced ACE2 access and infection. The authors also correlate their findings with human ALI-organoid cultures, proving biological relevance. The single-cell RNA seq data visualized in diffusion maps matched with the experimental data, where the expected expression of each mucin subtype was shown to be distributed as expected in the ALI and Calu3 cell types. The discussion places the findings of the paper in relevant context – where care is taken to explain that while MUC1 may provide a novel therapeutic target to limit SARS-CoV-2 infection, treatments that alter the homeostasis of this glycoprotein during the course of infection may alter other important functions that MUC1 plays in immune regulatory responses to the infection, but clearly this cs-mucin plays an important role in COVID severity.

This is a high quality manuscript which clearly answers an important and currently high impact research question.

Reviewer #2: In this manuscript, Chatterjee et al. investigate the role of tethered mucins and, specifically, their terminal glycans during SARS-CoV-2 infection. They utilize both Calu3 monolayer cultures and primary airway epithelial organoids (air-liquid interface cultures) along with various enzymatic treatments to remove glycosylated extracellular mucin domains, specific sialic acids, or fucose molecules to determine whether these mucins/glycans may promote adherence, or act as a barrier to infection. In conclusion, the authors propose a model in which mucins block access to ACE2 and thereby restrict infection. While the paper is well-written, the majority of the paper is spent characterizing mucin expression after StcE treatment, which remains somewhat unclear due in part to the limited availability of antibodies that detect specific domains of specific mucins. Further, it is unclear why the authors have chosen to focus much of the paper on Calu3 cells which lack cilia and fail to express all the major tethered airway mucins (e.g. MUC16). The most exciting result is found in Figure 5, where StcE treatment of airway cultures yields a significant increase in infection. However, the paper does not resolve which tethered mucin(s) may be responsible for the barrier function. This, in conjunction with the fact that several aspects of the work have been investigated before (e.g. ACE2 and mucin expression in tissue and airway models, impact of NA treatment on infection, etc.), significantly limits the impact and novelty of the study.

Reviewer #3: This submission reports findings that transmembrane mucins restrict SARS-CoV-2 entry. The report has convincing data that demonstrates mucin interference with virus-cell binding. Mucins have been investigated in many laboratories for their effects on virus infections. The submission also provides findings on the roles of sialic acid, fucose and heparin as SARS-CoV-2 susceptibility factors. While these latter findings were negative (enzymatic removal had no effect on infection), they may be significant in addressing the credibility of previous reports claiming that these carbohydrates are involved in SARS-CoV-2 entry.

**Part II – Major Issues: Key Experiments Required for Acceptance**
Please use this section to detail the key new experiments or modifications of existing experiments that should be absolutely required to validate study conclusions.

Generally, there should be no more than 3 such required experiments or major modifications for a "Major Revision" recommendation. If more than 3 experiments are necessary to validate the study conclusions, then you are encouraged to recommend "Reject".

Reviewer #1: There are no major issues for this manuscript that I can tell.

Reviewer #2: • The authors note in the introduction that MUC16 is associated with cilia (citing a review from Hattrup and Gendler published in 2008). However, more recent work by Kesimer et al. (doi: 10.1038/mi.2012.81) has suggested MUC16 localizes to goblet cells.

The reference was added to the introduction. We also performed experiments to address the expression of MUC16 in different cell types and observed expression in goblet cells and some ciliated cells in air-liquid grown primary epithelium. These results were added as Figure 7C-E.

• In Figure S1 and 1, the authors detect little MUC16 in Calu3 cells and fail to detect MUC4 on the surface of Calu3 cells. They suggest that MUC4 has intracellular localization; however, it may also be that the antibody used does not detect the mature glycosylated form of MUC4. Can the authors confirm whether or not this is the case?

As the reviewer is obviously aware of, mucins remain very challenging when it comes to antibody detection. We used anti-MUC4 clone 8G7 clone (sc-53945, Santa Cruz Biotechnology) which is directed against an epitope in the extracellular repeat domain and is one of the commonly used MUC4 antibodies. With respect to the issue of different glycoforms, this antibody was shown to work in WB on highly glycosylated MUC4 (smear visible so seems to detect different glycoforms of MUC4) (product website). Therefore, we think that in this case we have a good antibody to detect MUC4, but it cannot be excluded that different glycoforms are poorly detectable. For a future study, we may use some of the antibodies described in by Kesimer et al (https://doi.org/10.1038/mi.2012.81) that further looked into recognition of different glycoforms of respiratory MUC4 by different MUC4 antibodies.

Further, if the data are in fact correct and there is no MUC16 or MUC4 expression on the surface of Calu3 cells, then these cells do not seem to be a good model system in which to interrogate the impact of tethered mucins on SARS-CoV-2 infection.

To investigate the role of TM mucins other than MUC1, we performed infection experiments with the organoid-derived airway cultures. As for the use of Calu-3 cells, we think they are an excellent model to investigate the specific contribution of MUC1.

• At line 138 the authors note a partial loss of MUC1-ED detection after NA treatment, but indicate MUC1 was still detectable on the surface of cells by IF and thus, the western results are likely due to a change in epitope recognition. While not a focus of the manuscript, the IF data here appear to be missing. Also, given the partial loss, some MUC1 expression on the cell surface would be expected, and since the IF data does not appear to have been quantified in any way, the rationale for interpreting the western blot in this way is unclear.

The reviewer is right that we did not provide the immunofluorescence data here and that the text is too speculative. We added a dotblot to the figure to reinforce that MUC1 is still present after neuraminidase treatment (Fig 2D). As this is indeed not a main focus of the manuscript, we removed the reference to the IF.

• The results in Fig 2H are a bit confusing – the loss of MUC1 detection is obvious after StcE treatment, but X409 binding does not appear altered. Shouldn’t X409 binding be lost with StcE treatment? The authors note that StcE targets an O-glycosylated serine-threonine motif that is virtually absent in non-mucin proteins (line 123). If this is true, then what is X409 binding to in Fig 2H on the surface of Calu3 beyond MUC1? On a related note, please separate the channels in the confocal microscopy (Figure 2H and 2I) as some of the signal is difficult to see in the merge.

We adjusted the text to explain the experiment and results better. Also, separate channels were included for the confocal microscopy in 2H and 2I.

• Similarly, in Fig 4D,E, why doesn’t X409 staining co-localize with MUC1 as it seems to do in Fig 2H? The colocalization with MUC16 in Fig 4E is also not convincing. It seems that both MUC1 and MUC16 should react with X409.

We have now added the separate channels for Figure 2H, more clearly demonstrating that X409 and MUC1 poorly colocalize. We observe the same result with our air-liquid grown primary cultures (Figure 4D). We added a quantification of MUC1/X409 and MUC16/X409 colocalization on two donors and indeed see a much stronger colocalization with MUC16. Based on predicted glycosylation patterns of MUC1 and MUC16, we hypothesize that StcE has only limited recognition sites in MUC1 and many in MUC16. Because of this amplifying effect, staining of MUC16 with X409 is most likely much stronger. We see similar preferential binding of E447D to mucins containing multiple recognition sites in western blot analysis (not in this paper).

• It is unclear why the authors return to Calu3 cells in Figure 6. Why not incubate the airway cultures with Fc-tagged spike? A similar experiment was recently successfully performed using quantum dot-conjugated SARS-CoV-2 RBD in airway cultures to assess binding (doi: 10.1016/j.cell.2022.11.030).

Thank you for the suggestion. We performed the experiments with the airway cultures and spike binding for two donors and included these as Figure 6C.

• In Fig 6E, the authors conclude that spike-positive pseudoviral particles preferentially bound to MUC1-negative regions. This conclusion appears to be based on one single region of spike staining, and is thus not well supported.

The reviewer is right that this conclusion was not well supported by the provided data. For this reason, we have removed Figure 6E.

• The authors conclude that mucins block access to ACE2 and thereby restrict infection, and the current data in Calu3 cells would suggest this block is primarily due to MUC1. How do the authors fit this model with their data showing that MUC1 and ACE2 only partially co-localize on the apical surface of Calu3 and with published work in human airway cultures placing ACE2 on the apical membrane of ciliated cells (<https://doi.org/10.1128/JVI.79.24.15511-15524.2005>) or on cilial shafts (DOI: 10.1038/s41467-020-19145-6) while MUC1 is found on microvilli, predominantly on non-ciliated cells?

We agree that it is an essential step forward for the field to establish the exact localization and expression the different transmembrane mucins and ACE2 in the different models and cell types. To start addressing this point, we invested in an additional set of experiments. We performed proximity ligation experiments to investigate the interaction between ACE2 and MUC1 in Calu-3 cells and confocal microscopy to determine expression of MUC1 and MUC16 in different cell types in the primary airway cultures. The results of these experiments are included in Figure 7B-G. We also included a paragraph in the discussion to include these results and the suggested papers.

• Overall, the manuscript would be strengthened by including image quantification or colocalization analysis, as well as additional means of mucin removal (e.g. knockout of individual or all tethered mucins).

Yes, we had the same line of reasoning and tried to generate Calu-3 MUC1 knockout cells lines. While this approach was successful in our intestinal cells, the Calu-3 cells proved to be very challenging to manipulate. We invested many months in different knockout approaches but remained unsuccessful. Therefore, we need to make do with the mucinase removal for the current story but we think our combination of cell line and primary tissues experiments make a convincing story.

Additional quantification and colocalization analysis was added in Figure 4 and Figure S3.

Reviewer #3: 1. One concern with this submission is that many of the findings have been reported previously, particularly in PMID: 35879412 (ref 29), which communicates experiments and results strikingly similar to those in this submission. While these prior works were discussed on lines 280-300, further improvements in the submission would come with clear indication of the findings that are new, with clearer statements of their significance in advancing current understanding of mucins in SARS-CoV-2 infection.

The reviewer is right that the paper by Biering et al reports similar findings as our own. The discussion is adjusted to emphasize the significance of our findings.

2. Interpretation of findings and the Fig 6F model appeal to the close proximity of MUC1 with ACE2. While there are data showing MUC1 – ACE2 coexpression, the discussion and model in Fig 6F could be further supported with higher resolution imaging. Might proximity ligation assays generate more convincing evidence of MUC-ACE2 colocalization?

We thank the reviewer for this nice suggestion. We performed PLA experiments for MUC1-ED with ACE2 and MUC1-SEA with ACE2 and both combinations resulted in positive PLA signal demonstrating close proximity of the two proteins. The results of these experiments are included as Figure 7B.

3. S1B-Fc is far smaller than SARS-CoV-2 particles. Is S1B-Fc a suitable probe for TM MUC-mediated steric interference with ACE2 binding, given that it is small and possibly more penetrant into the mucus layer than virions?

Yes, indeed the spike purified protein is much smaller than the viral particles so it might be the case that we are underestimating the steric hindrance capacity of TM mucins for intact virions. However, we do see in other studies in our group that TM mucins also reduce binding of antibodies to surface receptors. Experiments in which we determine binding of virus particles to the surface would be an interesting future line of investigation.

(Also, a minor question about the SARS-pseudotyped VSV that was used to probe ACE2 binding – is this a VSV that has GFP incorporated into the virus particles, or is it a VSV that expresses GFP after S-mediated transduction? Probably it is the former, but perhaps it should be overtly specified as the virus is used as a probe for ACE2 binding.)

The reviewer is raising a good point here that we did not consider previously. We are indeed using a pseudovirus that expresses GFP after S-mediated transduction. In this light, we are unsure what GFP signal we observe in Figure 6D. For this reason, we decided to remove this piece of data from the manuscript as it does not alter the conclusion.

4. While the discussion section is interesting and informative, there could be mention and discussion of PMID: 36580912, which shows restricted SARS-CoV-2 cell binding being overcome by attachment at the tips of cilia that extend beyond the mucus layers.

Thank you for the suggestion, we included the paper in the discussion.

**Part III – Minor Issues: Editorial and Data Presentation Modifications**
Please use this section for editorial suggestions as well as relatively minor modifications of existing data that would enhance clarity.

Reviewer #1: Line 174: "viral" infection - consider changing the word to pseudovirus

Changed to pseudovirus.

Figure 6A: the labels within the photos blend in with positive staining - consider changing these labels to white.

The labels of this figure (now 7A) were adjusted.

"Authentic" SARS-CoV-2: It is appreciated that the authors are showing the difference between pseudovirus and whole virus experiments, but "Authentic" isn't really the nomenclature to use. Perhaps just use the word "pandemic"?

We would like to continue using the term “authentic” virus to distinguish these experiments from our pseudovirus experiments.

Given the advent of evolution of variants of concern (VOCs) which have many mutations in the spike protein with somewhat altered receptor binding affinity/specificity and dependency on different proteases for Spike cleavage, it would be good to indicate that the isolate used by this study matches the earlier or original 'outbreak' virus, versus the emerged VOCs.

A line was added in the materials and methods to include this information.

Reviewer #2: -Line 76, “Influenza” should not be capitalized

This was adjusted.

-The term “organoids” does not seem appropriate when referring to human airway cultures on transwells.

We have changed this terminology to “human organoid-derived airway cultures”.

-What does the “N” distinction in Fig 1A refer to? Nasal?

Yes, indeed this is referring to “nasal”. We added this to the legend of the figure.

-Line 117, Fig 1F should be Fig 1E

This was adjusted.

-The language is confusion at times around the activity of StcE. For example, in some places it seems the enzyme is removing the entire extracellular domain (e.g. line 125) vs. the associated glycans (e.g. line 129). This should be clarified throughout.

The text was adjusted to emphasize that StcE recognizes an *O*-glycosylated serine-threonine motif but that it cleaves the mucin polypeptide backbone.

-Fig 3E states “authentic SARS-CoV-2” yet in the text at line 185, this seems to refer to pseudoparticles, and there is no Fig 3F as referred to on line 192.

Thank you for pointing this out. We mistakenly were referring to a figure that was moved elsewhere. The sentence talking about figure E is now removed and F is changed to E.

-Line 255, do you mean Fig 6E instead of Fig 4E?

Yes, that is correct. But these data are removed according to the reviewers suggestions.

-Line 302 should read “initial infection”

This was changed.

-please check the y-axis in Figure S2C as the values seem incorrect.

Thank you, we adjusted to Y axis.

Reviewer #3: (No Response)

PLOS authors have the option to publish the peer review history of their article ([what does this mean?](https://journals.plos.org/plospathogens/s/editorial-and-peer-review-process#loc-peer-review-history)). If published, this will include your full peer review and any attached files.


If you choose “no”, your identity will remain anonymous but your review may still be made public.


**Do you want your identity to be public for this peer review?** For information about this choice, including consent withdrawal, please see our [Privacy Policy](https://www.plos.org/privacy-policy).

Reviewer #1: **Yes:** Julie McAuley

Reviewer #2: No

Reviewer #3: No
