# Supplementary material for: Glycosylated extracellular mucin domains protect against SARS-CoV-2 infection at the respiratory surface
Source: PLoS Pathog. 2023 Aug 10;19(8):e1011571. doi: 10.1371/journal.ppat.1011571 (PMC10464970; doi:10.1371/journal.ppat.1011571)
Supplement: Attachment — Submitted filename: Response to reviewers_190723.docx [file ppat.1011571.s006.docx]

**Response To Reviewers**

We thank the reviewers for carefully reading through our manuscript and making these final suggestions. We adjusted the manuscript and hope this improved version will be accepted for publication.

**Part II – Major Issues: Key Experiments Required for Acceptance**

Reviewer #1: No major issues identified

Reviewer #2: Line 252 – the StcE- and E447D-treated conditions in Fig 6C look very similar, and only one image of each condition / donor is shown. Quantification here across multiple experiments/images is needed here to support the conclusions.

We agree with the reviewer that quantification of S1B binding across multiple experiments was lacking in the manuscript. As prompted by the reviewer, we performed the quantification in two different donors for multiple experiments. We observe considerable variation between both donors, experiments and conditions. It is possible that viral binding in a stratified epithelial cell culture containing a multitude of different cell types (such as our ALI cultures) is more complex than cell lines such as Calu-3 cells and allows additionally binding of S1B independent of ACE2 binding. In addition, differences in the genetic background between donors is likely to affect S1B binding. Future studies should determine virus-receptor binding in organoid cultures and the potential role of mucins in preventing this interaction in more detail.

Because we can no longer draw a clear conclusion from these experiments, we decided to remove Figure 6C from the manuscript. We added a discussion of our observations and changed our conclusions accordingly in lines 252-261. We believe that despite removing Figure 6C, the role of transmembrane mucins in blocking ACE2 binding is still supported by Figures 6A and B using Calu-3 cells.

Figure 7A/B – what is the frequency of ACE2/MUC1 double positive cells? This again seems important to quantify to support their model.

We agree with the reviewer and quantified the data from Figure 7A and B. In 7A, 35% were positive for both ACE2 and MUC1 and in the proximity ligation assay 40% of cells were found to be positive. These percentages are included in the text, line 267 and 272.

Reviewer #3: (No Response)

**Part III – Minor Issues: Editorial and Data Presentation Modifications**
Please use this section for editorial suggestions as well as relatively minor modifications of existing data that would enhance clarity.

Reviewer #1: All minor issues identified in my earlier review have been fixed.

Reviewer #2: The authors note that MUC4 and MUC5AC could not be stained in Fig 1D (line 115), indicating these mucins are intracellular. Given that MUC5AC is known to be a secreted mucin and in light of previous data by other groups showing MUC4 expression on the cell surface in airway cultures (PMID: 22929560), a cautionary statement should be included here regarding the limitations of antibodies used, staining protocol, or expression levels of these targets in these cells. As it reads currently, it appears that the authors believe these mucins are simply not expressed on the surface or secreted in this model which is unlikely.

We have included a cautionary statement in line 116-117 about the limitations of the antibodies.

Please note in the methods what controls were used in IF experiments (e.g. secondary antibody only?) to set the thresholds.

Indeed, this was missing from the materials and methods section. We used incubations with secondary antibody only as a negative control for all IF experiments and included this information in the methods section in line 962-963.

Line 188. Figure 3E appears to be authentic virus in the figure, not pseudoparticles as noted here. There is also no figure 3F as referred to on line 196.

Thank you for pointing out this mistake, we corrected it.

Line 331 should read “while ciliated cells”

This mistake was corrected (line 337).

Line 337 states that MUC1 was “found to be highly expressed on microvilli of basal epithelial cells.” I believe this should state that MUC1 was found on microvilli AND on basal epithelial cells (as demonstrated in Figure 5 of the referenced publication).

Yes, indeed. This mistake was corrected (line 343).

Reviewer #3: (No Response)
